# Improved Growth Media for Isolation and Identification of Fish Pathogenic *Tenacibaculum* spp.

**DOI:** 10.3390/microorganisms13071567

**Published:** 2025-07-03

**Authors:** Erwan Lagadec, Ingeborg Emilie Berg Kahrs, Kathleen Frisch, Henrik Duesund, Are Nylund, Sverre Bang Småge

**Affiliations:** 1 Fish Disease Research Group, Department of Biological Sciences, University of Bergen, 5007 Bergen, Norway; ingeborg.kahrs@uib.no (I.E.B.K.); are.nylund@uib.no (A.N.); sverre.smage@cermaq.com (S.B.S.); 2 Cermaq Canada, Campbell River, BC V9W 2C2, Canada; kathleen@frisch.pro; 3 Cermaq Group AS, 0277 Oslo, Norway; henrik.duesund@cermaq.com

**Keywords:** Atlantic salmon, *Tenacibaculum*, tenacibaculosis, winter ulcers

## Abstract

Standard blood agar medium with 2% NaCl (BAS) and Marine Agar (MA) are commonly used in bacteriological investigations of winter ulcers in farmed Atlantic salmon (*Salmo salar* Linnaeus) in Norway and allow easy recovery of *Moritella viscosa* based on its characteristic viscous colonies and β-hemolytic activity. However, the recent increase in cases of winter ulcers involving *Tenacibaculum* spp. and the potential emergence of *T. maritimum* due to rising temperatures highlight the need for improved methods of isolation and identification. Indeed, the recovery of *Tenacibaculum* spp. from outbreaks of winter ulcers or tenacibaculosis can be challenging. Despite the development of several agar media over the years to overcome this issue, such as *Flexibacter maritimus* medium (FMM), it remains difficult to differentiate *Tenacibaculum* species. We evaluated the growth dynamics and phenotypic characteristics of 13 bacterial isolates commonly associated with ulcer outbreaks on five different agar media, including two new formulations: kanamycin-supplemented marine blood agar for the selective isolation of *Tenacibaculum* spp. (KABAMA) and general blood agar for marine bacteria (BAMA). These new media facilitate the identification of *Tenacibaculum* spp., including *T. maritimum*, by distinguishing colonies based on their specific color, shape, and hemolytic activity.

## 1. Introduction

Tenacibaculosis is a significant disease in many economically important marine fish species, including salmonids, and is caused by bacteria of the genus *Tenacibaculum* [1,2]. This disease is characterized by frayed fins, tail rot, mouth erosion, skin lesions, and ulcers [1]. Several *Tenacibaculum* spp., including *Tenacibaculum dicentrarchi*, *T. finnmarkense*, *T. maritimum*, and *T. piscium*, have been shown to induce tenacibaculosis in challenge studies [3,4,5,6,7,8].The psychrotrophic *T. finnmarkense* is mostly associated with tenacibaculosis at lower seawater temperatures [7,9], while increased prevalence and severity of tenacibaculosis associated with *T. maritimum* have been reported at higher temperatures, above 15 °C [1,10]. The clinical manifestations of diseases caused by these bacteria are also different in that *T. finnmarkense* and *T. dicentrarchi* typically affect non-scaled skin, while *T. maritimum* may affect the entire body, including the gills, producing a distinct slimy bacterial mat on body surfaces [2,10,11,12,13].

Ulcerative skin diseases occurring in lower temperature seawater (e.g., ‘winter ulcers’) are a major health and welfare problem for the global salmonid-farming industry [14,15] and have been reported in Norway since the late 1980s [16,17,18]. While the bacterium *Moritella viscosa* is considered the main etiological agent of ‘winter ulcers’, several other bacterial species contribute to ulcerative diseases. The most frequently associated bacteria are *Aliivibrio wodanis* and *Tenacibaculum* spp. [18,19]. Several studies have shown that *T. finnmarkense* genomovar (gv) *finnmarkense* is the dominant *Tenacibaculum* species associated with ulcerative disease in farmed salmon in Norway [6,7,9]. In the Norwegian salmon farming industry, the recovery of *Tenacibaculum* spp. from field outbreaks of ulcerative diseases using standard nutrient agar media has proven challenging despite the presence of a large number of bacteria in the ulcerative lesions with *Tenacibaculum* morphology (long slender rods) [18]. This is likely due to the historical use of blood agar media containing 1.5–2.0% NaCl (BAS) as the main medium for investigating bacterial diseases in saltwater salmon life stages. The increased use of Marine Agar (MA) (e.g., Difco 2216 or Zobell 2216), which contains sea salts and therefore mimics the natural composition of seawater, has improved the recovery of *Tenacibaculum* spp. from field samples [18,20]. Some *Tenacibaculum* spp. grow only in the presence of varying concentrations of sea salts and exhibit poor growth with NaCl alone [7,18,21]. Despite these improvements, recovering *Tenacibaculum* spp. from skin lesions or ulcers in farmed Atlantic salmon (*Salmo salar* Linnaeus) remains challenging, particularly in cases of mixed infections [18].

MA supports the growth of a wide range of heterotrophic marine bacteria, which can make the isolation of *Tenacibaculum* spp. challenging, as these may be outcompeted, inhibited, or overgrown by fast-growing marine bacteria like *Aliivibrio*, *Vibrio*, *Alteromonas,* and *Pseudoalteromonas* [22,23,24,25,26,27]. To circumvent this issue for *T. maritimum*, the specific medium *Flexibacter maritimus* medium (FMM) was developed to limit the growth of other heterotrophic marine bacteria (e.g., *Vibrio* spp., *Alteromonas* spp.) [28]. In addition to improving the recovery of *T. maritimum* from mixed infections, FMM may help indicate the serotype of a certain isolate [25,28]. Marine Shieh’s Selective Medium (MSSM), originally developed for isolating *Flavobacterium columnare* [29], has also proven effective for recovering *Tenacibaculum* spp., including *T. maritimum*, particularly in New Zealand, where it is routinely used for disease surveillance [27,30]. The intrinsic filamentous morphology of *T. maritimum* and its ability to adhere to substrates pose challenges for experimental infection models and can hinder the recognition of *T. maritimum* colonies [10,31,32].

Another option for creating a selective nutrient agar is to add an antimicrobial agent to the medium, which limits the growth of extraneous bacteria. For *Tenacibaculum*, several different agents have proven effective, most of which belong to the class of aminoglycoside antibiotics (e.g., kanamycin and neomycin), to which *Tenacibaculum* spp. appear to be innately resistant [20,22,24,26,33].

This study investigated the benefits, including the ease of colony selection, of a modified nutrient agar medium for the isolation and growth of *Tenacibaculum* spp. The growth of 11 bacterial strains in the genus *Tenacibaculum* and two bacterial species commonly associated with ‘winter ulcer’ outbreaks in Norwegian salmonids was compared on five different agar media.

## 2. Materials and Methods

### 2.1. Nutrient Agar Media Preparation

BAMA was formulated as shown in Table 1. All components, except defibrinated sheep blood, were mixed and sterilized at 121 °C for 15 min. The agar medium was then cooled down to 50 °C before blood was aseptically added. Selective agar (KABAMA) was achieved by adding kanamycin to a final concentration of 50 µg mL^−1^ concurrent with the addition of blood (Table 1) to improve the primary isolation of *Tenacibaculum* spp. by restricting the growth of faster-growing extraneous bacteria. FMM (Condalab, Madrid, Spain) and MA (Difco 2216, BD Difco, Franklin Lakes, NJ, USA) were prepared according to the manufacturers’ protocols. BAS was ordered from the Norwegian Veterinary Institute (http://vetinst.no, accessed on 30 June 2025). Plates were stored at 4 °C and used before the expiration date.

### 2.2. Comparison of Bacterial Growth on Five Different Agar Media

The growth of 13 bacterial strains was compared using BAMA, KABAMA, BAS, FMM, and MA agar media. The study included 11 *Tenacibaculum* strains: *T. adriaticum* strain B390^T^, *T. dicentrarchi* strain NCIMB 14598^T^, *T. finnmarkense* gv. *finnmarkense* strain HFJ, *T. finnmarkense* gv. *ulcerans* strain TNO010^T^, *T. maritimum* strains NCIMB 2154^T^, CAN 15-1, NLF-15, and Ch-2402, *T. ovolyticum* strain NCIMB 13127^T^, *T. piscium* strain TNO020^T^, and *T. soleae* strain LL04 12.1.7^T^.

*Tenacibaculum*-type strains were obtained from the National Collection of Industrial, Food, and Marine Bacteria (http://ncimb.com, accessed on 1 June 2024). Upon receipt, the identity of each bacterial strain was verified by 16S rRNA gene sequencing and compared with reference sequences. *T. maritimum* strains CAN 15-1, Ch-2402, and NLF-15 were isolated from Atlantic salmon in Canada [26], Chile [34], and lumpsuckers (*Cyclopterus lumpus*, Linnaeus) in Norway [35], respectively. *T. finnmarkense* gv *finnmarkense* strain HFJ was isolated from skin lesions of diseased Atlantic salmon [7]. Two strains of bacterial species commonly associated with ‘winter ulcers’ were also included in the study: *Moritella viscosa* type strain NCIMB 13584^T^ and an isolate of *Aliivibrio wodanis* recovered from a farmed Atlantic salmon suffering from skin disease in Norway. Bacterial inocula were cultured directly from cryo stocks. After thawing, an inoculum of 400 µL was added to either 30 mL of Marine Broth (MB, Difco) in a 50 mL tube or, for the *T. maritimum* strains, to 1 L of MB in a 2 L Erlenmeyer flask. Cultures were incubated at 16 °C and 140 rpm for approximately 48 h or at 16 °C and 230 rpm for approximately 74 h for the *T. maritimum* strains. The most probable number (MPN) method was used to estimate the number of cells per inoculum [36]. A 30 µL aliquot of the bacterial culture was subsequently streaked in triplicate onto each agar medium and incubated at different temperatures. Relative growth and colony morphology were observed and documented daily for seven days. The β-hemolytic activity of the strains was readily observed by visualizing the plates under a light source. Images were captured using a Nikon D80 digital camera with an AF-S Micro Nikkor 60 mm 1:2.8G ED lens (Nikon Corporation, Tokyo, Japan) in a portable photo studio. Each culture was performed in triplicates.

The Details of the bacterial strains included in the study, incubation temperatures, and estimated number of bacteria streaked onto the agar plates for the tests are included in Table 2.

### 2.3. Phenotypic Characterization

To thoroughly characterize the morphology of the 11 *Tenacibaculum* strains on the different agar media, three plates per medium (BAMA, KABAMA, FMM, and MA) were inoculated with a bacterial suspension to yield approximately one to 20 colonies per plate. After inoculation, the plates were incubated at 16 °C, and daily observations were recorded over seven days to document growth patterns and phenotypic characteristics, such as colony color, shape, margin, and elevation. The number of colonies counted and the average colony diameter are detailed in Supplementary Material Table S1.

## 3. Results

### 3.1. Growth Comparison

**Figure 1:**
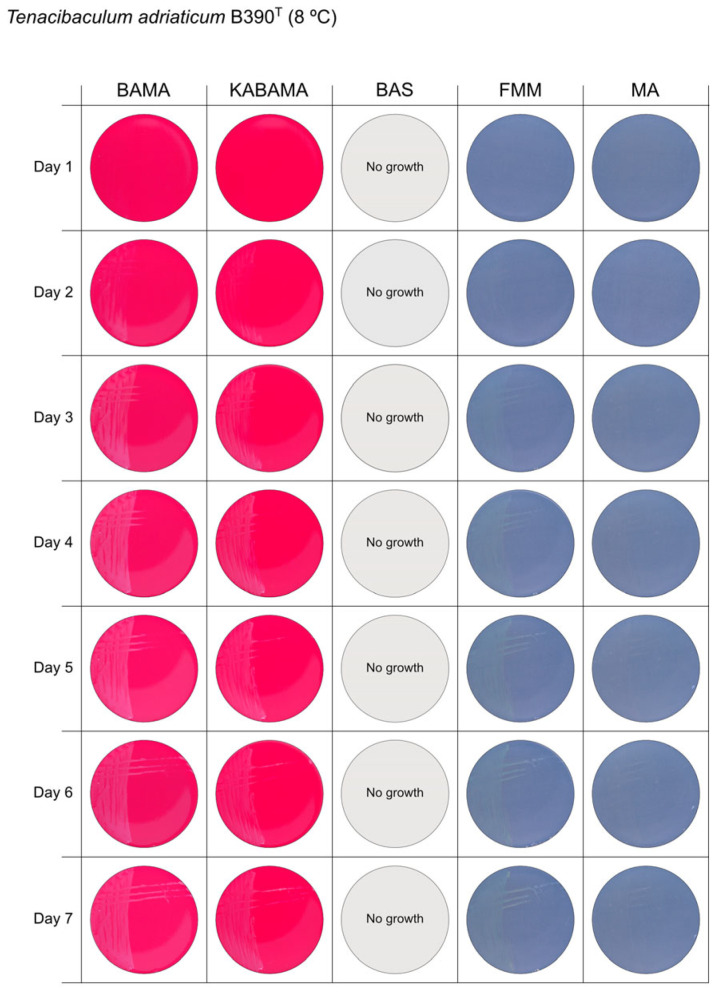
Seven-day growth of *Tenacibaculum adriaticum* strain B390^T^ at 8 °C on BAMA, KABAMA, BAS, FMM, and MA.

**Figure 2:**
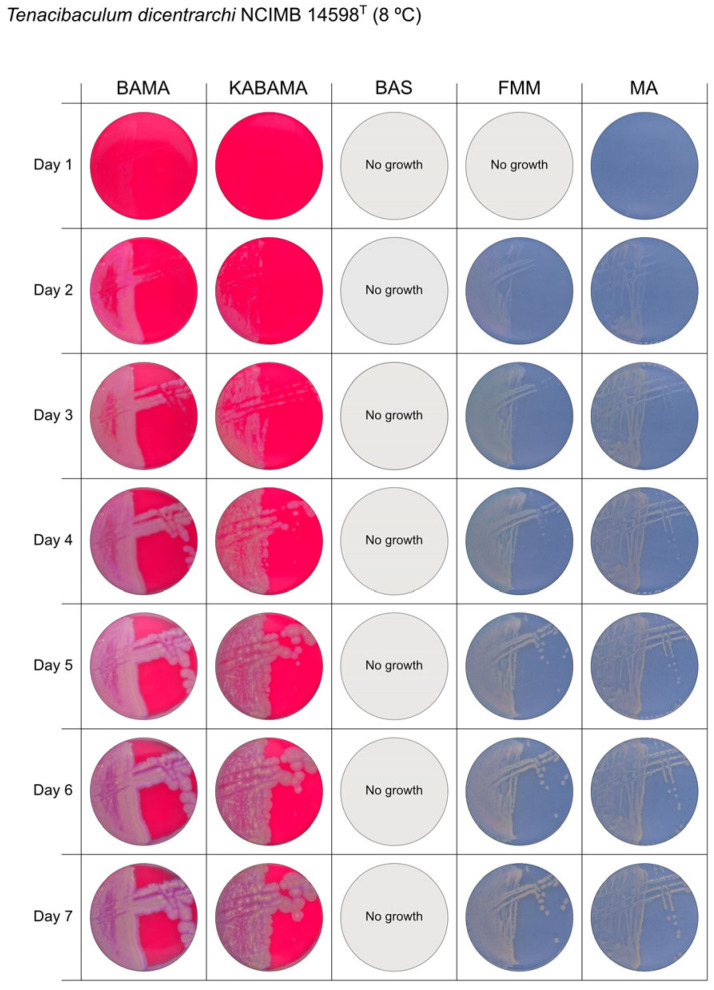
Seven-day growth of *Tenacibaculum dicentrarchi* strain NCIMB 14598^T^ at 8 °C on BAMA, KABAMA, BAS, FMM, and MA.

**Figure 3:**
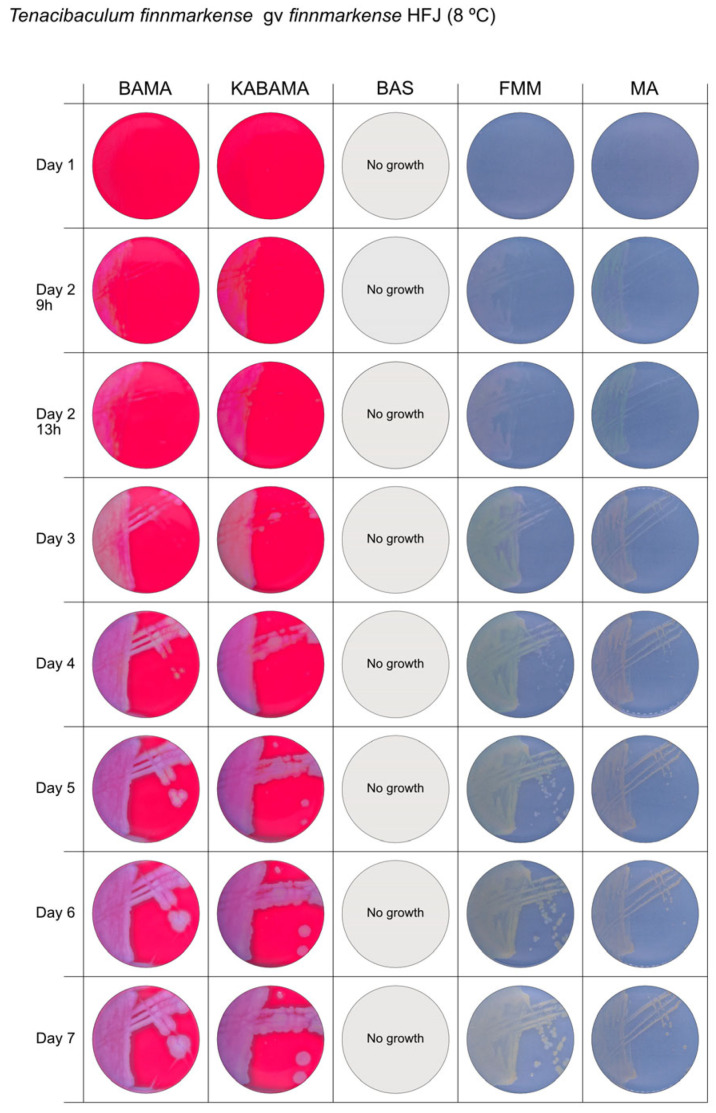
Seven-day growth of *Tenacibaculum finnmarkense* genomovar *finnmarkense* strain HFJ at 8 °C on BAMA, KABAMA, BAS, FMM, and MA.

**Figure 4:**
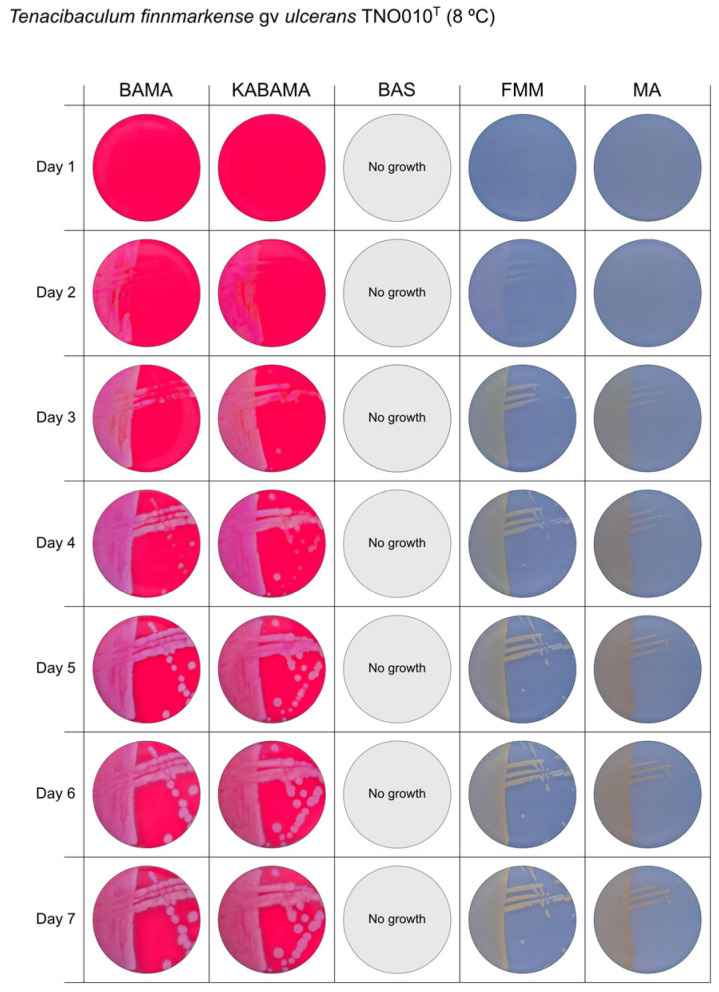
Seven-day growth of *Tenacibaculum finnmarkense* genomovar *ulcerans* strain TNO010^T^ at 8 °C on BAMA, KABAMA, BAS, FMM, and MA.

**Figure 5:**
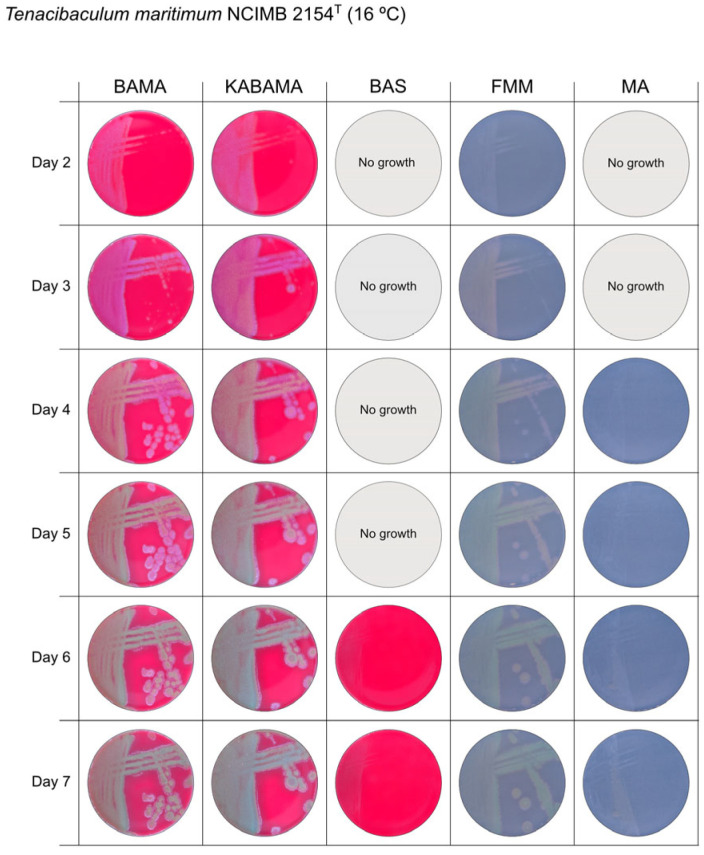
Seven-day growth of *Tenacibaculum maritimum* strain NCIMB 2151^T^ at 16 °C on BAMA, KABAMA, BAS, FMM, and MA.

**Figure 6:**
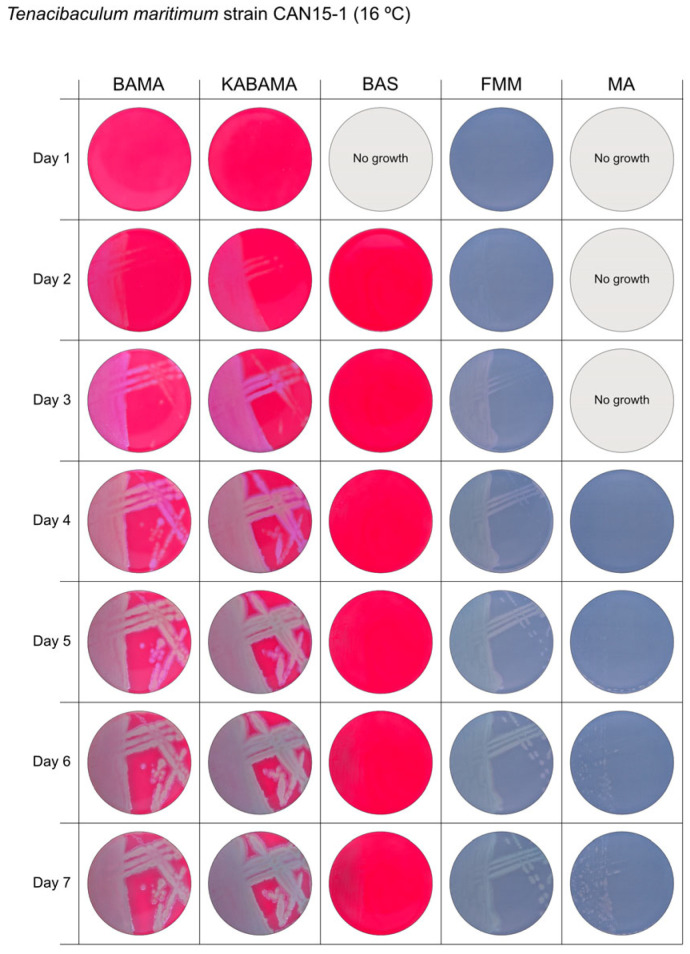
Seven-day growth of *Tenacibaculum maritimum* strain CAN 15-1 at 16 °C on BAMA, KABAMA, BAS, FMM, and MA.

**Figure 7:**
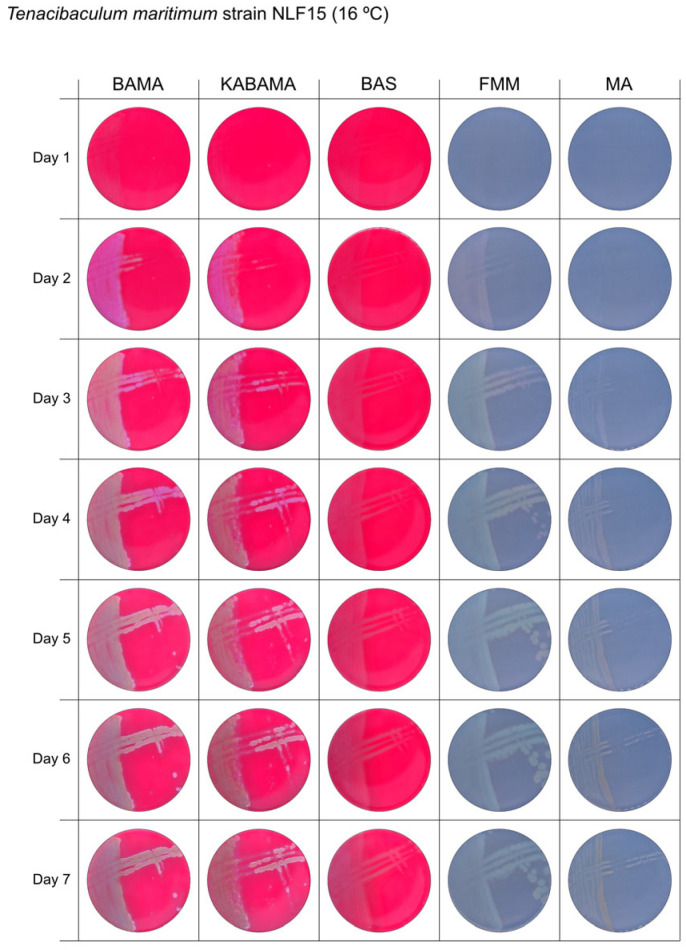
Seven-day growth of *Tenacibaculum maritimum* strain NLF-15 at 16 °C on BAMA, KABAMA, BAS, FMM, and MA.

**Figure 8:**
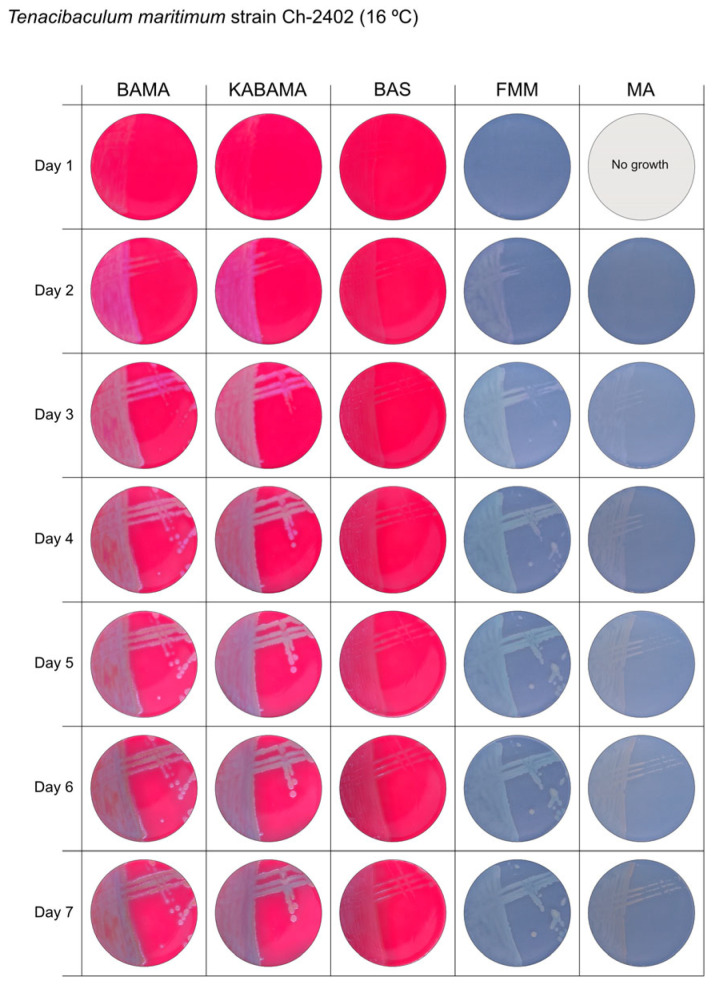
Seven-day growth of *Tenacibaculum maritimum* strain Ch-2402 at 16 °C on BAMA, KABAMA, BAS, FMM, and MA.

**Figure 9:**
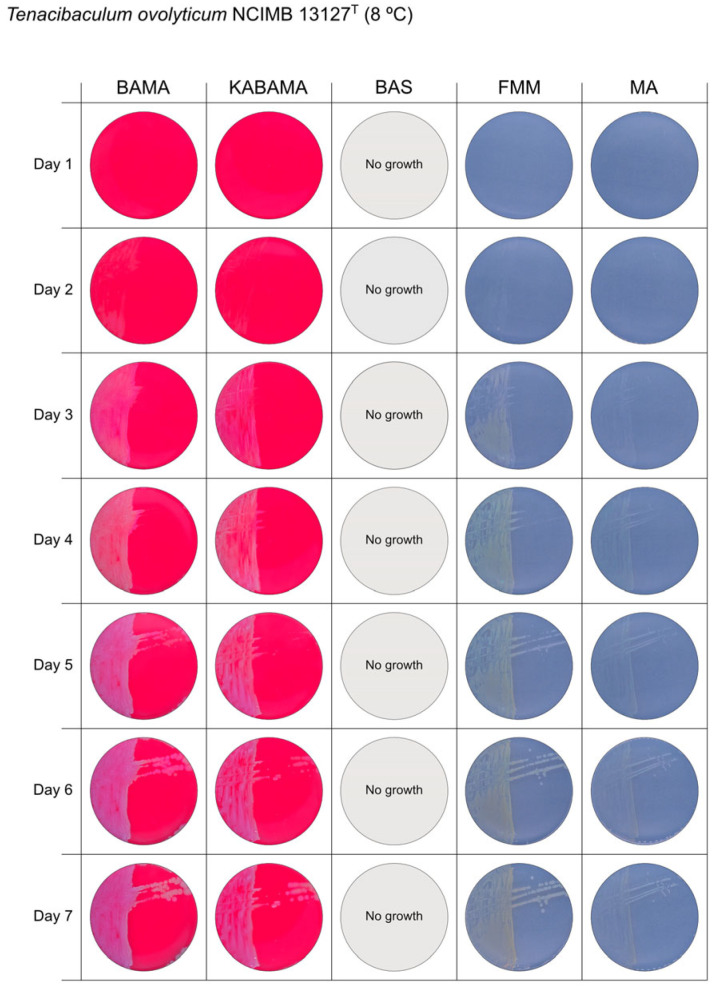
Seven-day growth of *Tenacibaculum ovolyticum* strain NCIMB 13127^T^ at 8 °C on BAMA, KABAMA, BAS, FMM, and MA.

**Figure 10:**
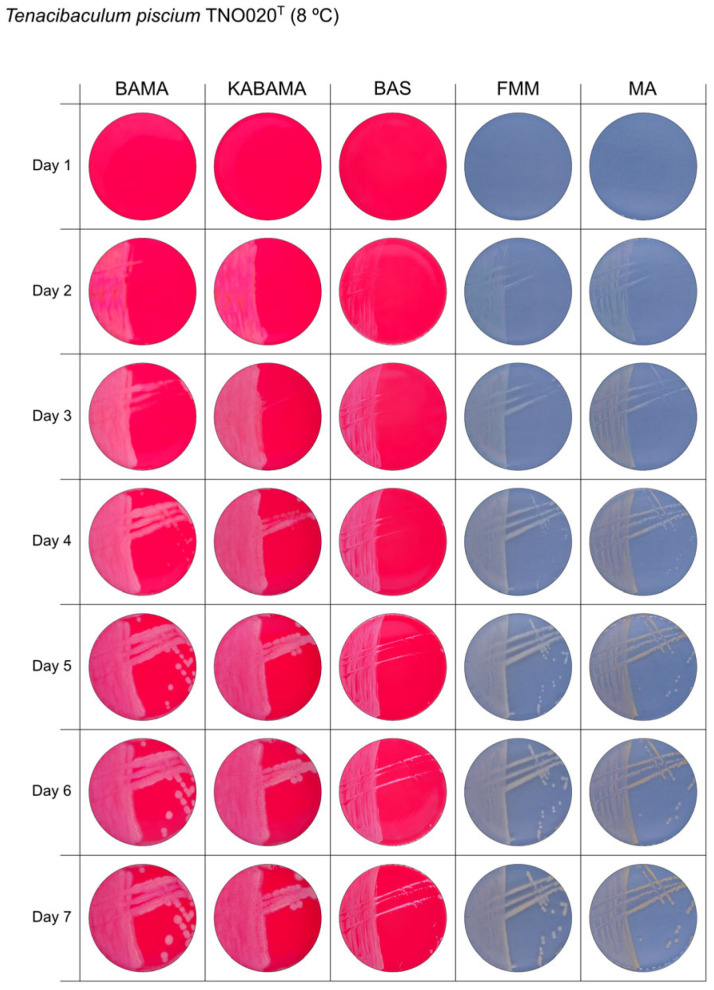
Seven-day growth of *Tenacibaculum piscium* strain TNO020^T^ at 8 °C on BAMA, KABAMA, BAS, FMM, and MA.

**Figure 11:**
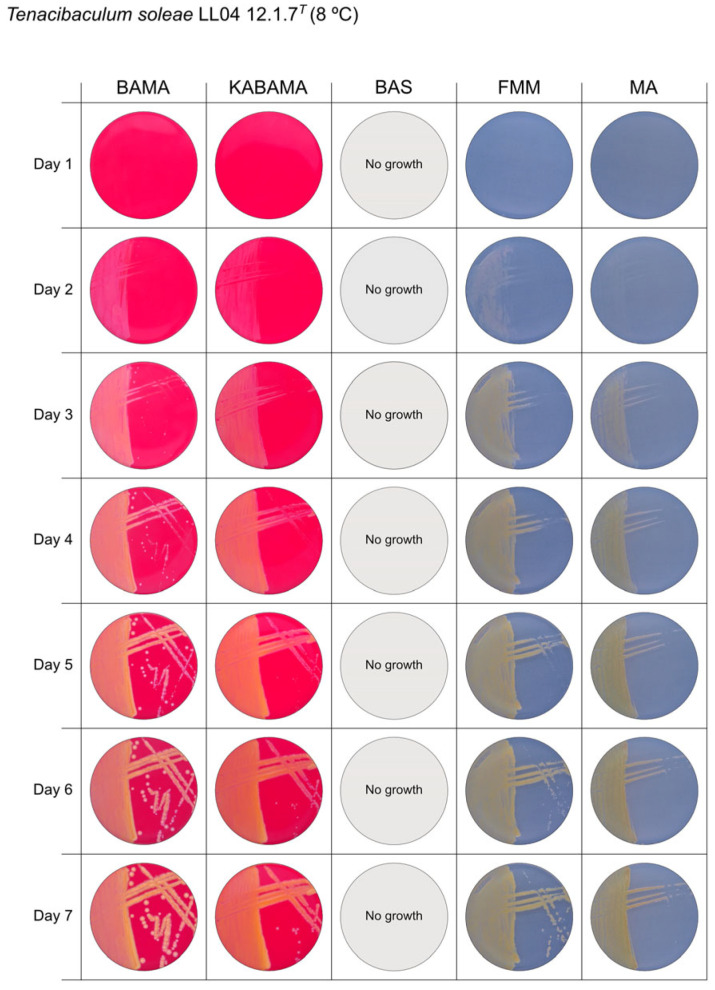
Seven-day growth of *Tenacibaculum soleae* strain LL04 12.1.7^T^ at 8 °C on BAMA, KABAMA, BAS, FMM, and MA.

**Figure 12:**
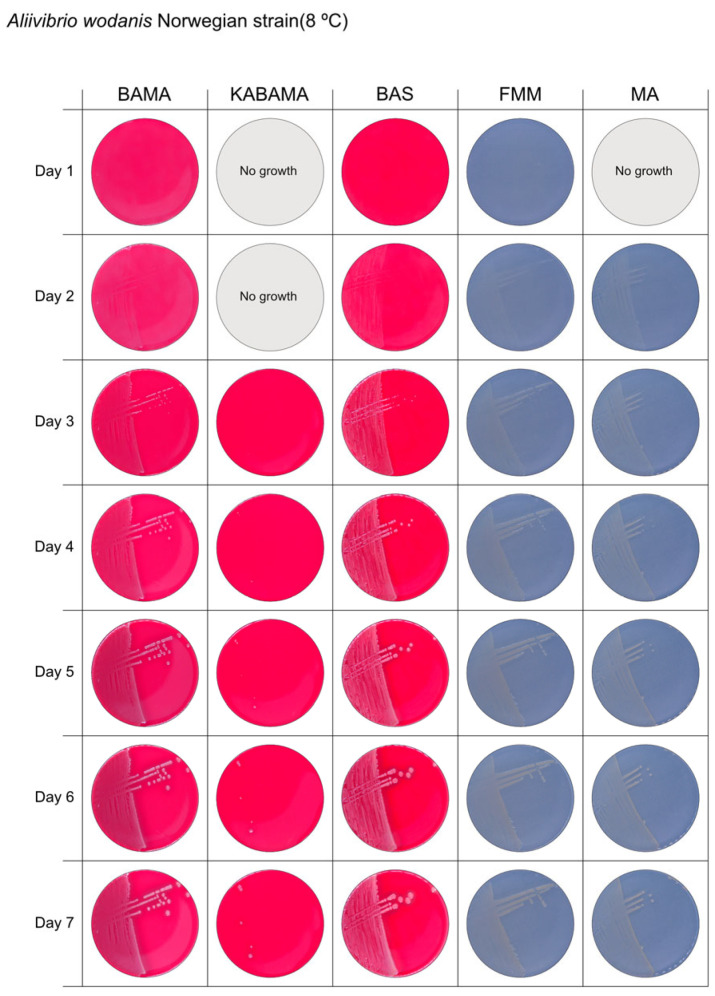
Seven-day growth of *Aliivibrio wodanis* Norwegian field strain at 8 °C on BAMA, KABAMA, BAS, FMM, and MA.

**Figure 13:**
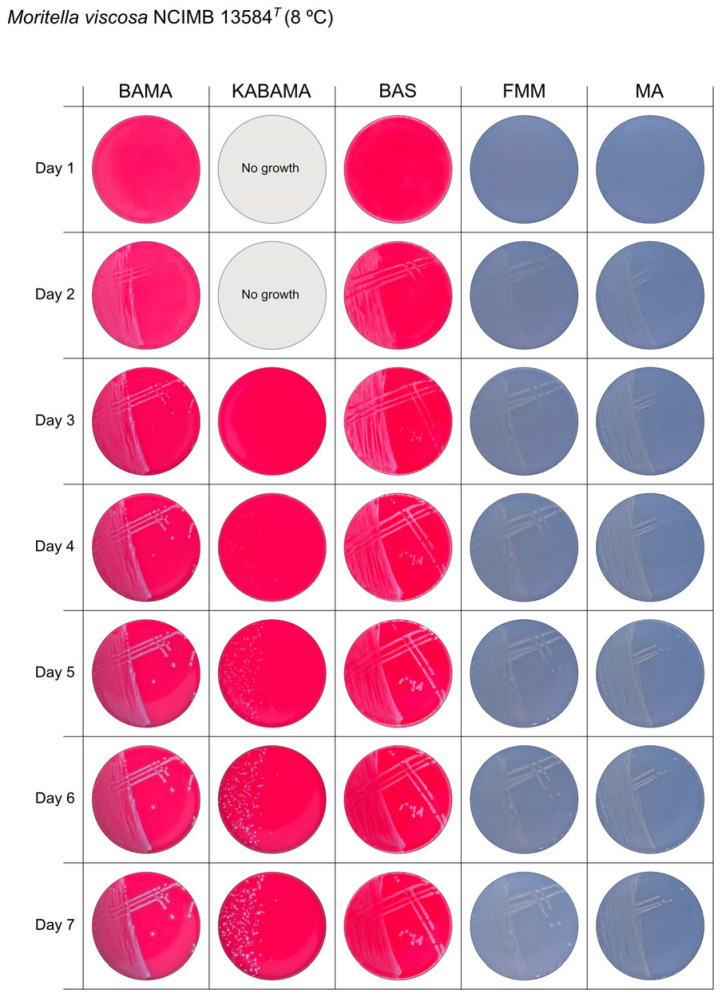
Seven-day growth of *Moritella viscosa* strain NCIMB 13584^T^ at 8 °C on BAMA, KABAMA, BAS, FMM, and MA.

All *Tenacibaculum* strains included in this study demonstrated stronger growth on BAMA and KABAMA, and to a lesser extent on FMM, than on MA and BAS under identical conditions (Figure 1, Figure 2, Figure 3, Figure 4, Figure 5, Figure 6, Figure 7, Figure 8, Figure 9, Figure 10 and Figure 11). Notably, the four *T. maritimum* strains exhibited slow and sparse growth on both MA and BAS media (Figure 5, Figure 6, Figure 7 and Figure 8). Among the other *Tenacibaculum* strains, none grew on BAS except for *T. piscium* strain TNO020^T^ (Figure 10). In contrast, the *A. wodanis* field strain and *M. viscosa* strain NCIMB 13584^T^ grew well on BAS, BAMA, MA, and FMM but not on kanamycin-supplemented KABAMA (Figure 12 and Figure 13, respectively).

### 3.2. Phenotypical Characteristics

**Figure 14:**
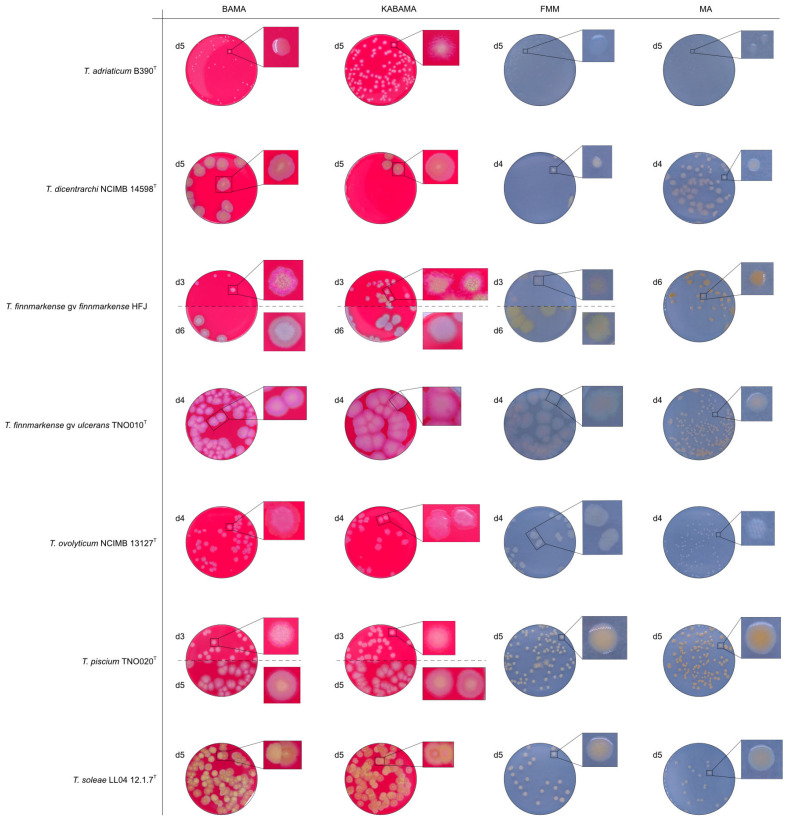
Comparison of colony characteristics for *Tenacibaculum adriaticum* strain B390^T^, *T. dicentrarchi* strain NCIMB 14598^T^, *T. finnmarkense* genomovar *finnmarkense* strain HFJ, *T. finnmarkense* genomovar *ulcerans* strain TNO010^T^, *T. ovolyticum* strain NCIMB 13127^T^, *T. piscium* strain TNO020^T^, and *T. soleae* strain LL04 12.1.7^T^ grown at 16 °C on BAMA, KABAMA, FMM, and MA. ‘d’ indicates days of growth. Dilution factors used for plating may differ between agar media for the same strain, to achieve optimal colony density for imaging.

**Figure 15:**
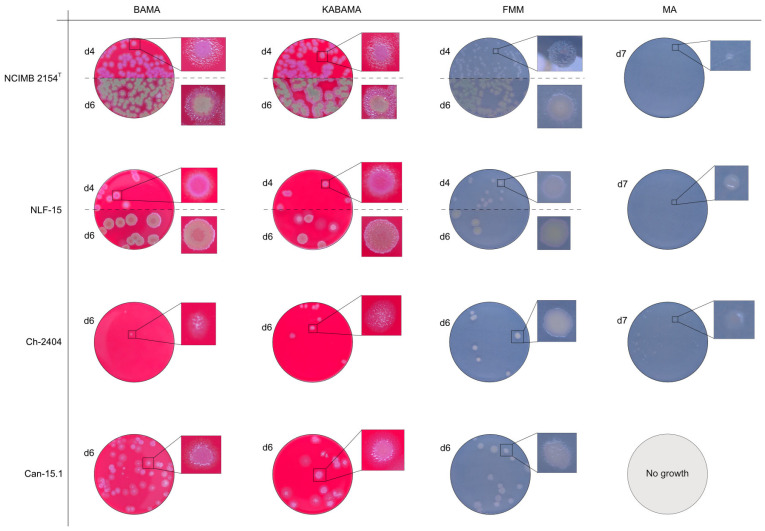
Comparison of colony characteristics for *Tenacibaculum maritimum* strains NCIMB 2151^T^, CAN 15-1, NLF-15, and Ch-2402 grown at 16 °C on BAMA, KABAMA, FMM, and MA. ‘d’ indicates days of growth. Dilution factors used for plating may differ between agar media for the same strain, to achieve optimal colony density for imaging.

The *Tenacibaculum* strains displayed distinct morphological characteristics on BAMA and KABAMA, allowing for clear differentiation based on size, shape, margin, color, iridescence, and hemolytic properties (Figure 14 and Figure 15, Table 3). On both BAMA and KABAMA, a marked change in color between the center and margin of the colonies was observed for *T. dicentrarchi* strain NCIMB 14598^T^, *T. finnmarkense* gv *finnmarkense* strain HFJ, *T. finnmarkense* gv *ulcerans* strain TNO10^T^, *T. piscium* strain TNO020^T^, and *T. soleae* strain LL04 12.1.7^T^. These strains, along with *T. maritimum* strain NCIMB 2154^T^, exhibited varying degrees of iridescence on BAMA and KABAMA. In contrast, differentiation between the strains of *Tenacibaculum* on MA and FMM was challenging, as the colonies displayed a more uniform appearance with relatively similar shapes and colors.

Generally, *Tenacibaculum* spp. colonies often showed greater spreading activity, with larger and often merged colonies on BAMA and KABAMA than on MA, FMM, and BAS.

Colonies of *T. maritimum* grown on BAMA and KABAMA displayed characteristic ruggedness and uneven margins, contrasting with the smooth colony surfaces and often even margins observed for all the other bacteria tested (Figure 15). These noticeable characteristics were less distinct on FMM and not visible on MA. *T. maritimum* strains NCIMB 2154^T^, Ch-2402, and NLF-15 developed a dark green coloration after prolonged incubation on BAMA and KABAMA (and, to a lesser extent, on FMM), which was not observed in any other *Tenacibaculum* spp. tested. The four *T. maritimum* strains exhibited sufficient phenotypic characteristics on BAMA and KABAMA for clear differentiation.

β-hemolytic activity was evaluated on blood-supplemented agar media BAMA, KABAMA, and BAS, and it was found that *T. dicentrarchi* strain NCIMB 14598^T^, *T. finnmarkense* gv. *finnmarkense* strain HFJ, and all *T. maritimum* strains exhibited strong β-hemolytic activity. In contrast, *T. ovolyticum* strain NCIMB 13127^T^ exhibited weak β-hemolytic activity, while no β-hemolysis was observed for *T. adriaticum* strain B390^T^, *T. finnmarkense* gv *ulcerans* strain TNO10^T^, *T. piscium* strain TNO020^T^, and *T. soleae* strain LL04 12.1.7^T^. Notably, *M. viscosa* strain NCIMB 13584^T^ and *A. wodanis* field strain also demonstrated strong β-hemolytic activity.

A summary comparison of the five media used for the cultivation and differentiation of *Tenacibaculum* spp. is included in Supplementary Material Table S2.

## 4. Discussion

This study demonstrates that KABAMA and BAMA are more specific and effective for *Tenacibaculum* growth than MA and BAS. Previous studies comparing growth media for *Tenacibaculum* similarly reported limited growth on MA compared to FMM and MSSM [27,28]. Although FMM is also suitable for *Tenacibaculum* growth, strain differentiation is less distinct on FMM than on BAMA and KABAMA due to the absence of blood in its composition.

The temperatures chosen for the documentation of bacterial growth (Figure 1, Figure 2, Figure 3, Figure 4, Figure 5, Figure 6, Figure 7, Figure 8, Figure 9, Figure 10, Figure 11, Figure 12, Figure 13, Figure 14 and Figure 15) were selected to best illustrate the colony morphology under conditions representative of each strain’s optimal growth range; however, this may have introduced a bias that should be considered when interpreting visual comparisons.

Most *Tenacibaculum* spp. did not grow on BAS, as BAS only contains NaCl (2%), whereas *Tenacibaculum* spp. generally require sea salt in their growth media. Both BAMA and KABAMA included coral pro salt, which has a higher calcium content and may support better growth of *T. maritimum*; calcium-rich tissues are known to be preferred colonization sites for this bacterium [43]. Given the potential for batch-to-batch variability in commercial coral pro salt, future work should investigate the effects of defined calcium concentrations in the medium. This could contribute to the development of a standardized, chemically defined medium for *Tenacibaculum* spp.

The non-*Tenacibaculum* strains tested in this study exhibited limited growth on KABAMA medium. In contrast, both *Flavobacterium* and *Tenacibaculum* species in the Family Flavobacteriaceae are intrinsically resistant to kanamycin, as noted in the species descriptions for both genera. The addition of 50 μg/mL kanamycin to agar media has been shown to selectively support the growth of these bacteria [26,44].

Several *Tenacibaculum* species associated with tenacibaculosis outbreaks in aquaculture exhibit strong β-hemolytic activity (e.g., *T. dicentrarchi*, *T. finnmarkense*, *and T. maritimum*). In contrast, non-hemolytic strains of *T. adriaticum* and *T. soleae* have not been associated with disease in farmed Atlantic salmon. Whole-genome sequencing has confirmed that *Tenacibaculum* type strains displaying β-hemolytic activity contain genes encoding hemolysins [45,46,47]. Interestingly, although *T. soleae* also possesses hemolysin genes [48], no β-hemolytic activity was observed in this study. Additionally, the non-hemolytic species *T. piscium* has been associated with skin ulcer development in several sea-farmed fish species [6], and scale loss and frayed fins were observed in dead fish during a bath-challenge experiment [8]. Although β-hemolytic bacteria are typically considered pathogenic and β-hemolysis is often a strong predictor of bacterial pathogenicity [49], this correlation warrants further investigation of *Tenacibaculum* spp. isolated from outbreaks associated with skin lesions or ulcers in Norwegian farmed Atlantic salmon.

Several *Tenacibaculum* strains exhibit variable motility characteristics depending on their growth medium. *T. dicentrarchi* strain NCIMB 14598^T^, *T. finnmarkense* genomovars, *T. maritimum* strain NLF-15, and to a lesser extent *T. piscium* strain TNO020^T^, *T. soleae* strain LL04 12.1.7^T^, and *T. maritimum* strain NCIMB 2154^T^, show increased motility on BAMA and KABAMA compared to growth on MA and BAS. A previous study by Pazos and Santos [28] demonstrated that MA does not induce gliding motility as effectively as other agar media, such as FMM. Gliding motility has been observed in *Flavobacterium columnare* when cultured in low-nutrient content [50], which may explain the enhanced gliding motility observed in FMM, containing half the yeast extract of MA. In contrast, BAMA and KABAMA had nutrient contents equivalent to MA, suggesting that the addition of blood and/or Red Sea Salt in the agar media could stimulate the observed gliding motility (evident from the colony shapes) in the *Tenacibaculum* strains listed above. Blood components may activate virulence genes under conditions that mimic fish ulcer environments [51,52]. Genes encoding virulence factors, including hemolysins and the T9SS-mediated secretion system, are important for gliding motility and have been identified in both *T. finnmarkense* and *T. dicentrarchi* [6,18]. *T. maritimum* also contains genes encoding hemolysins [10]; however, it exhibited unique gliding motility characteristics in this study, with smaller colonies and a rougher surface than other *Tenacibaculum* spp. in this study, making it easily distinguishable. The size difference in *T. adriaticum* strain B390^T^ observed on BAMA or KABAMA may result from the presence of kanamycin and potentially be related to antibiotic resistance expression, particularly if gliding motility genes are present on the same plasmid, as suggested by Schroeder and Brooks [53]. Johansen, Catón [54] further noted that gliding motility is crucial for structural coloring in colonies within the Flavobacteriaceae family, which may explain why BAMA and KABAMA, which support effective gliding motility, induced intense bacterial colony coloration.

In the Norwegian salmon industry, BAS and MA are the most commonly used media for investigating skin lesions and ulcerations. The historic reliance on BAS for diagnosing such outbreaks may partly explain why *Tenacibaculum* spp. have not been recognized as significant pathogens associated with these diseases. Since most *Tenacibaculum* spp. do not grow on media supplemented solely with NaCl, BAS tends to favor the growth of other bacteria present in skin lesions and ulcers, such as *M. viscosa* and *A. wodanis*, which grow well on this medium. This likely introduced bias in earlier bacteriological investigations, potentially overestimating *M. viscosa* as the primary etiological agent in skin lesion outbreaks. The challenges associated with detecting or isolating certain *Tenacibaculum* sp., particularly *T. maritimum*, and the resulting underestimation of their role in disease development have already been reported, notably in New Zealand [27,55].

The use of KABAMA provides a selective advantage for recovering pathogenic *Tenacibaculum* spp. associated with skin lesions and ulcers in Atlantic salmon. This medium is particularly beneficial for isolating *T. maritimum*, which typically grows more slowly than other bacteria found in skin lesions, including other *Tenacibaculum* species. Both KABAMA and BAMA revealed specific characteristics of *T. maritimum* colonies, such as distinct ruggedness and uneven margins, β-hemolytic activity, and distinct green coloration upon prolonged incubation. These characteristics aid in the accurate identification and recovery of *T. maritimum* strains in the future. Additionally, KABAMA and BAMA enhanced the recovery of other *Tenacibaculum* spp., revealing typical colony characteristics like smooth surfaces, β-hemolytic activity, and distinct color differences between strains. Field investigations confirmed the benefits of using KABAMA, which facilitated the rapid detection and isolation of *Tenacibaculum* colonies (see Supplemental Material, Figures S1–S3).

Overall, the use of KABAMA and BAMA enables the effective differentiation and identification of *Tenacibaculum* spp. associated with skin lesions and ulcer outbreaks in farmed Atlantic salmon and can reveal the presence of concurrent *Tenacibaculum* spp. in the same sample. Identifying multiple *Tenacibaculum* strains from a single outbreak provides valuable insights into the genetic diversity of *Tenacibaculum* spp.

## Figures and Tables

**Table 1:** List of components and formula for 1 L of BAMA and KABAMA agar media.

Component	Quantity
Peptone from animal tissue (P7750, Merck, Darmstadt, Germany)	5.0 g
Yeast extract (Y1625, Merck)	1.0 g
Coral pro salt (Red Sea Fish Farm Ltd., Herzliya, Israel)	37.0 g
Bacteriological agar (A5306, Merck)	15.0 g
Milli-Q water (Sigma-Aldrich, Burlington, MA, USA)	950 mL
Sterile defibrinated sheep blood (SR0051E, Thermo Fisher, Waltham, MA, USA)	50 mL
* Kanamycin 50 mg ml^−1^ (Merck)	1 mL

* For KABAMA.

**Table 2:** List of the 13 bacterial strains included in this study. The number of cells per agar plate was estimated from the most probable number (MPN) of the inoculum.

Bacterial Strain	Cell Number per Agar Plate	Incubation (°C)	Reference
*T. adriaticum* B390^T^	>1.8 × 10^8^	8, 16	[37]
*T. dicentrarchi* NCIMB 14598^T^	1.21 × 10^7^	8, 16	[38]
*T. finnmarkense* genomovar *finnmarkense* HFJ	2.9 × 10^6^	4, 8, 16	[7]
*T. finnmarkense* genomovar *ulcerans* TNO010^T^	2.52 × 10^7^	4, 8, 16	[6]
*T. maritimum* NCIMB 2154^T^	2.31 × 10^6^	12, 16, 25	[39]
*T. maritimum* CAN 15-1	1.14 × 10^8^	12, 16, 25	[26]
*T. maritimum* NLF-15	4.8 × 10^7^	12, 16, 25	[35]
*T. maritimum* Ch-2402	1.21 × 10^8^	12, 16, 25	[34]
*T. ovolyticum* NCIMB 13127^T^	9.3 × 10^7^	4, 8, 16	[40]
*T. piscium* TNO020^T^	1.96 × 10^7^	4, 8, 16	[6]
*T. soleae* LL04 12.1.7^T^	1.8 × 10^8^	8, 16	[41]
*Aliivibrio wodanis* Norwegian strain	1.41 × 10^6^	8, 16	This study
*Moritella viscosa* NCIMB 13584^T^	5.7 × 10^7^	8, 16	[42]

**Table 3:** Hemolytic activity and morphological characteristics of the 13 strains included in the study. All bacteria were grown at 16 °C. Hemolytic activity is described as ‘ND’ (not detectable on the medium), ‘-’ (γ-hemolysis), ‘+’ (α-hemolysis or weak β-hemolysis), ‘++’ (strong β-hemolysis), ‘+++’ (very strong β-hemolysis).

Bacterial Strain	Agar Medium	Hemolytic Activity	Morphological Characteristics
*T. adriaticum* B390^T^	BAMA	-	Small grey-purple, round colonies, entire margins
	KABAMA	-	Medium-size, yellow round colonies with purple irregular margins
	BAS	ND	No growth
	FMM	ND	Small, round, grey (faint yellow) colonies with entire margins
	MA	ND	Very small, round, and grey colonies with entire margins
*T. dicentrarchi* NCIMB 14598^T^	BAMA	++	Large umbonate colonies with yellow centers and purple undulating margins
	KABAMA	++	Purple iridescent colonies (2 first days), then large umbonate colonies with yellow center and purple undulating margins
	BAS	ND	No growth
	FMM	ND	Small yellow-grey entire colonies
	MA	ND	Medium-sized faint yellow, entire colonies
*T. finnmarkense* genomovar *finnmarkense* HFJ	BAMA	+++	Large, flat, very iridescent colonies, with green centers and purple irregular margins. Very large blue-green non-iridescent entire colonies after 96 h.
	KABAMA	+++	Large, flat, very iridescent colonies, with green centers and purple irregular margins. Very large blue-green non-iridescent entire colonies after 96 h.
	BAS	ND	No growth
	FMM	ND	Weak yellow-green color, slightly iridescent, large colonies with purple margins
	MA	ND	Medium-size green then yellow (after 72 h) entire colonies
*T. finnmarkense* genomovar *ulcerans* TNO010^T^	BAMA	-	Large purple iridescent undulating colonies. Purple-white without iridescence after 72 h
	KABAMA	-	Large purple iridescent undulating colonies. Purple-white without iridescence after 72 h
	BAS	ND	No growth
	FMM	ND	Large faint yellow-green color colonies with slight iridescent margins
	MA	ND	Small yellow entire colonies
*T. maritimum* NCIMB 2154^T^	BAMA	++	Medium-size purple flat colonies with irregular margins displaying important ruggedness. Large dark green with irregular margins and important ruggedness after 96 h
	KABAMA	++	Medium-size purple flat colonies with irregular margins displaying important ruggedness. Large dark green with irregular margins and important ruggedness after 96 h
	BAS	+	Slight growth after 120 h.
	FMM	ND	Small white-grey colonies with ruggedness. Medium-size yellow-grey colonies after 120 h
	MA	ND	Very small, faint, grey-yellow colonies after 96 h
*T. maritimum* CAN 15-1	BAMA	++	Purple medium-size colonies with important ruggedness and irregular margins.
	KABAMA	++	Purple medium-size colonies with important ruggedness and irregular margins.
	BAS	+++	Very slow growth of small, faint purple color colonies starting at 48 h
	FMM	ND	Grey medium-size colonies
	MA	ND	Very slow growth of small white-grey colonies displaying ruggedness
*T. maritimum* NLF-15	BAMA	++	Purple medium-size colonies with important ruggedness and irregular margins. Turn large green-yellow colonies with ruggedness after 96 h
	KABAMA	++	Purple medium-size colonies with important ruggedness and irregular margins. Turn large green-yellow colonies with ruggedness after 96 h
	BAS	+++	Slight growth of beige entire colonies
	FMM	ND	Medium-size colonies are grey−yellow colonies. Faint yellow colonies with light margin after 120 h
	MA	ND	Very small light colonies
*T. maritimum* Ch-2402	BAMA	+	Small purple colonies with irregular margins and strong ruggedness. Turn dark green after prolonged growth
	KABAMA	+	Medium-size purple colonies with irregular margins and strong ruggedness. Turn dark green after prolonged growth
	BAS	++	Very small white colonies
	FMM	ND	Medium-size faint yellow colonies with light margins
	MA	ND	Very small light colonies
*T. ovolyticum* NCIMB 13127^T^	BAMA	+ (weak)	Medium-size purple undulating colonies
	KABAMA	+ (weak)	Medium-size purple undulating colonies
	BAS	ND	No growth
	FMM	ND	Medium-large yellow−grey colonies
	MA	ND	Small white colonies
*T. piscium* TNO020^T^	BAMA	-	Medium-large yellow−white colonies with purple iridescent margins. Undulating entire colonies
	KABAMA	-	Medium-large yellow−white colonies with purple iridescent margins. Undulating entire colonies
	BAS	-	White small colonies
	FMM	ND	Small white-yellow colonies
	MA	ND	Small yellow colonies
*T. soleae* LL04 12.1.7^T^	BAMA	-	Medium-size yellow colonies with purple and green iridescent margins
	KABAMA	-	Medium-size yellow colonies with purple and green iridescent margins
	BAS	ND	No growth
	FMM	ND	Small yellow colonies
	MA	ND	Small colonies with a faint yellow-green color
*Aliivibrio wodanis* Norwegian strain	BAMA	++	Small white entire colonies
	KABAMA	++	Very slow growth of small white colonies
	BAS	++	Medium size white colonies
	FMM	ND	Small white entire colonies
	MA	ND	Small white entire colonies
*Moritella viscosa* NCIMB 13584^T^	BAMA	++	Small grey entire colonies with classic viscous characteristics
	KABAMA	++	Very slow growth of small white colonies with classic viscous characteristics
	BAS	++	Small white colonies with classic viscous characteristics
	FMM	ND	Small white entire colonies with classic viscous characteristics
	MA	ND	Small white entire colonies with classic viscous characteristics

## Data Availability

The original contributions presented in this study are included in the article. Further inquiries can be directed to the corresponding author.

## Supplementary Materials

The following supporting information can be downloaded at: https://www.mdpi.com/article/10.3390/microorganisms13071567/s1. Figure S1. Bacterial cultivation from a skin ulcer on an Atlantic salmon (*Salmo salar*) in a facility in Vestland, Norway. Figure S2. Bacterial cultivation from *Fucus vesiculosus* collected in Vestland, Norway. Figure S3. Bacterial cultivation from 2 filter tanks at a fish production site in Vestland, Norway. Table S1. Colony development of 11 *Tenacibaculum* bacterial strains on four different agar media. Table S2. Summary comparison of media performance for the cultivation and differentiation of *Tenacibaculum* spp. References [56,57] are cited in the Supplementary Materials.

## Abbreviations

The following abbreviations are used in this manuscript.

BAS	Blood agar medium supplemented with 2% NaCl
FMM	*Flexibacter maritimus* medium
MA	Marine Agar
