# Supplementary material for: Improved Growth Media for Isolation and Identification of Fish Pathogenic Tenacibaculum spp
Source: Microorganisms. 2025 Jul 3;13(7):1567. doi: 10.3390/microorganisms13071567 (PMC12300813; doi:10.3390/microorganisms13071567)
Supplement: Supplementary file 1 [file microorganisms-13-01567-s001.zip › Supplementary material_Figures S1-S3.pdf]

Supplementary Materials. Figures S1-S3

Bacterial growth was attempted from various sources on several agar media: Marine Agar (MA), blood agar medium with 2 % NaCl (BAS), *Flexibacter maritimum* medium (FMM), BAMA, and KABAMA.

Colonies were sub-cultured onto BAMA, and clonal bacterial cultures were preserved in a 1:1 Biofreeze freezing medium (Biochrom, Germany) and Marine broth (DIFCO 2216) in liquid nitrogen. Genomic DNA was extracted using DNeasy Blood & Tissue Kit (Qiagen), following the manufacturer's instructions. Bacterial identification was performed via 16S *rRNA* gene sequencing using primers 27F and 1518R [56]. *Tenacibaculum* strains were further included in a multilocus sequence analysis (MLSA) using primers targeting housekeeping genes as described in [57]. PCR products were run on 1% agarose gels stained with GelRed (Biotium, USA). Positive amplicons were purified using ExoCleanUp FAST (VWR) in a Veriti thermal cycler (Applied Biosystems) at 37°C for 5 min and 80°C for 10 min, and subsequently sent for Sanger sequencing (Azenta Life Sciences, Germany).

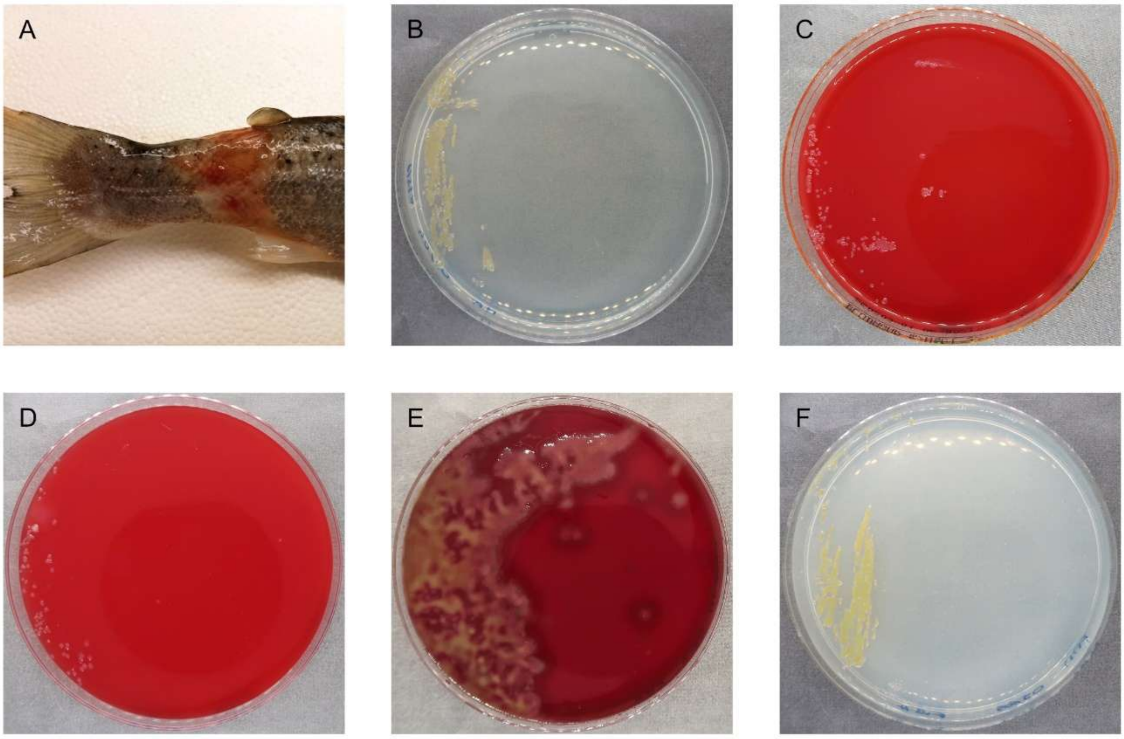

Figure S1. Bacterial cultivation from a skin ulcer on an Atlantic salmon (*Salmo salar)* in a facility in Vestland, Norway. Pictures show the ulcerated lesion on the fish skin (A), and bacterial growth after 72h of incubation at 8°C on MA (B), BAS (C), BAMA (D), KABAMA (E), and FMM (F). Notably, abundant and clearly identifiable growth of *Tenacibaculum dicentrarchi* was observed on KABAMA, while only *Maritomonas* sp. was detected on BAS. A mixed culture of *T. dicentrarchi* and *Maritomonas* sp. was observed on MA, BAMA, and FMM.

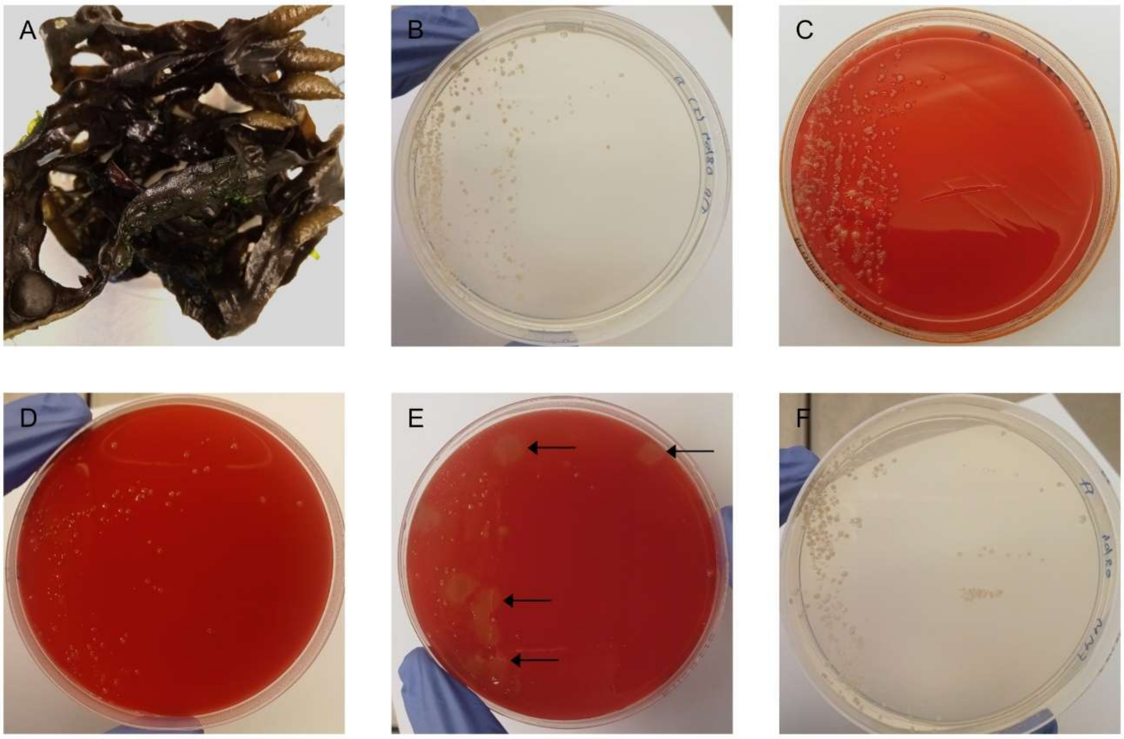

Figure S2. Bacterial cultivation from *Fucus vesiculosus* collected in Vestland, Norway. Pictures show the *F. vesiculosus* specimen (A), and bacterial growth after 48h of incubation at 8°C on MA (B), BAS (C), BAMA (D), KABAMA (E), and FMM (F). Several colonies of *Tenacibaculum dicentrarchi* were detected on KABAMA (indicated by arrows), while other media showed growth of distinct *Pseudoalteromonas* sp. colonies.

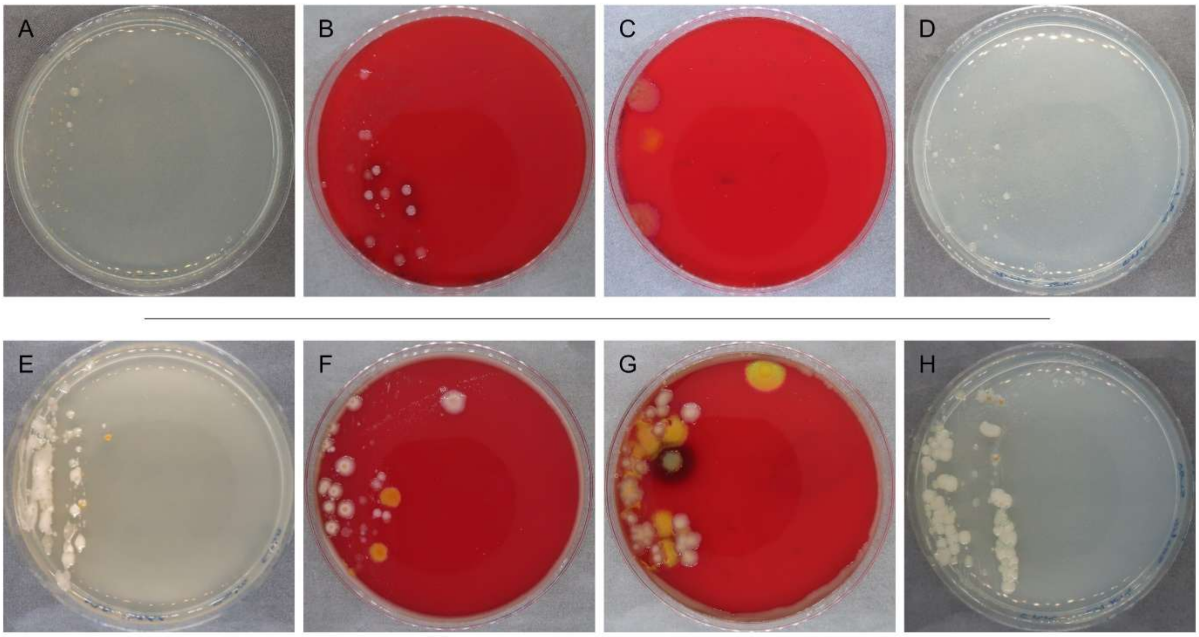

Figure S3. Bacterial cultivation from 2 filter tanks at a fish production site in Vestland, Norway. Pictures A-D show bacterial growth from 30 µL of water sampled from tank#1 after 72h of incubation at 8°C on MA (A), BAMA (B), KABAMA (C), and FMM (D). *Tenacibaculum dicentrarchi* and *T. ovolyticum* were isolated from KABAMA (C), while no *Tenacibaculum* growth observed on the other media after 72h. Pictures E-H show bacterial growth from a swab of tank#2 on MA (E), BAMA (F), KABAMA (G), and FMM (H) after 72h of incubation at 8°C. *T. ovolyticum* and a second *Tenacibaculum* species were isolated on KABAMA (G), and *Tenacibaculum* sp. was also isolated on BAMA (F). A mixed culture of *Halomonas* sp. and *Paraglaciecola* sp. might have inhibited *Tenacibaculum* growth on FMM.
