# Supplementary material for: Improved Growth Media for Isolation and Identification of Fish Pathogenic Tenacibaculum spp
Source: Microorganisms. 2025 Jul 3;13(7):1567. doi: 10.3390/microorganisms13071567 (PMC12300813; doi:10.3390/microorganisms13071567)
Supplement: Supplementary file 1 [file microorganisms-13-01567-s001.zip › Supplementary material_Table S1.pdf]

Supplementary Material. Table S1

Table S1. Colony development of 11 *Tenacibaculum* bacterial strains on four different agar media. Most Probable Number (MPN) values were determined for each bacterial strain prior to plating. *n.g.* = no growth observed at the time of measurement. DPP = days post-plating. SD = standard deviation. Colony diameter was measured on 10 colonies per plate; fewer colonies were measured when growth was limited. gv = genomovar. ^{T} = Type strain

Bacterial strain	Inoculum MPN (MPN/mL)	Dilution factor (plated)	Number of colonies on plate					Colony Diameter (mean ± SD, mm)				
			DPP	BAMA	KABAMA	FMM	MA	DPP	BAMA	KABAMA	FMM	MA
T. adriaticum strain B390^{T}	6,00E+09	10^{6}	6	40	126	12	4	5	1,9 ± 0,2	3,7 ± 0,8	2,0 ± 0,7	0,7 ± 0,2
T. dicentrarchi strain NCIMB 14598^{T}	4,05E+08	10^{5}	3	12	3	3	56	5	14,4 ± 2,5	12,3 ± 1,2	5,0 ± 0,0	6,6 ± 2,4
T. finnmarkense gv *finnmarkense* strain HFJ	6,75E+07	10^{4}	4	7	29	14	27	6	12,7 ± 1,5	10,7 ± 1,1	19,0 ± 2,5	3,5 ± 1,1
T. finnmarkense gv *ulcerans* strain TNO010^{T}	8,40E+08	10^{5}	3	70	20	3	4	4	10,1 ± 2,6	21,9 ± 3,7	16,4 ± 3,4	2,1 ± 0,8
T. maritimum strains NCIMB 2154^{T}	7,70E+07	10^{4}	3	182	161	202	5*	4	5,3 ± 0,6	5,9 ± 0,7	1,7 ± 0,6	n.g.
T. maritimum strain CAN 15-1	3,80E+09	10^{6}	5	9	16	16	0	4	5,7 ± 1,2	9,8 ± 1,9	6,2 ± 1,2	n.g.
T. maritimum strain NLF-15	1,60E+09	10^{6}	5	19	11	16	14*	4	6,8 ± 1,0	6,2 ± 0,4	3,8 ± 0,4	n.g.
T. maritimum strain Ch-2402	4,05E+09	10^{6}	6	13	9	62	56	6	1,1 ± 0,6	4,3 ± 0,9	5,7 ± 0,8	1,5 ± 0,4
T. ovolyticum strain NCIMB 13127^{T}	3,10E+09	10^{6}	5	52	22	15	83	4	4,6 ± 1,1	5,5 ± 0,8	6,9 ± 1,7	1,9 ± 0,2
T. piscium strain TNO020^{T}	6,55E+08	10^{6}	5	12	8	17	13	5	8,8 ± 2,1	9,2 ± 2,3	4,1 ± 0,6	3,4 ± 0,7
T. soleae strain LL04 12.1.7^{T}	6,00E+09	10^{7}	5	15	10	22	19	5	7,8 ± 1,7	7,9 ± 1,3	5,2 ± 0,7	4,1 ± 0,5

* Colonies appeared only after 7 days of growth. Diameter size not measurable (≤ < 0,1 mm)
