# Supplementary material for: Improved Growth Media for Isolation and Identification of Fish Pathogenic Tenacibaculum spp
Source: Microorganisms. 2025 Jul 3;13(7):1567. doi: 10.3390/microorganisms13071567 (PMC12300813; doi:10.3390/microorganisms13071567)
Supplement: Supplementary file 1 [file microorganisms-13-01567-s001.zip › Supplementary material_Table S2.pdf]

Supplementary Material. Table S2

Table S2. Summary comparison of media performance for the cultivation and differentiation of *Tenacibaculum* spp. +++ = strong support, ++ = moderate support, + = weak support, - = no growth

Medium	BAMA	KABAMA	FMM	MA	BAS
Growth of *Tenacibaculum* spp.*	+++	+++	+++	++	-
Growth of *Tenacibaculum maritimum***	+++	+++	+++	- / +	+
Growth of *Moritella viscosa* and *Aliivibrio wodanis****	+++	-	+++	++	+++
Visibility of β-hemolysis	yes	yes	no	no	yes
Phenotypic differentiation	+++	+++	+	-	-
Selectivity for *Tenacibaculum* (kanamycin)	no	yes	no	no	no

* Based on *T. adriaticum* strain B390^T, *T. dicentrarchi* strain NCIMB 14598^T, *T. finnmarkense* genomovar *finnmarkense* strain HFJ, *T. finnmarkense* genomovar *ulcerans* strain TNO010^T, *T. ovolyticum* strain NCIMB 13127^T, *T. piscium* strain TNO020^T, and *T. soleae* strain LL04 12.1.7^T

** Based on *T. maritimum* strains NCIMB 2154^T, CAN 15-1, NLF-15, and Ch-2402

*** Based on *Moritella viscosa* type strain NCIMB 13584^T and a Norwegian isolate of *Aliivibrio wodanis*.
